# Simulation of Soil Organic Carbon Effects on Long-Term Winter Wheat (*Triticum aestivum*) Production Under Varying Fertilizer Inputs

**DOI:** 10.3389/fpls.2018.01158

**Published:** 2018-08-08

**Authors:** Bhim B. Ghaley, Henk Wösten, Jørgen E. Olesen, Kirsten Schelde, Sanmohan Baby, Yubaraj K. Karki, Christen D. Børgesen, Pete Smith, Jagadeesh Yeluripati, Roberto Ferrise, Marco Bindi, Peter Kuikman, Jan-Peter Lesschen, John R. Porter

**Affiliations:** ^1^Department of Plant and Environmental Sciences, University of Copenhagen, Taastrup, Denmark; ^2^Wageningen Environmental Research, Wageningen University and Research, Wageningen, Netherlands; ^3^Department of Agroecology, Aarhus University, Tjele, Denmark; ^4^Institute of Biological and Environmental Sciences, University of Aberdeen, Aberdeen, United Kingdom; ^5^Information and Computational Sciences Group, The James Hutton Institute, Aberdeen, United Kingdom; ^6^Department of Agri-food Production and Environmental Sciences, University of Florence, Florence, Italy

**Keywords:** grain yield, DAISY model, nitrogen, plant available water, pedotransfer functions, long-term experiment, crop productivity

## Abstract

Soil organic carbon (SOC) has a vital role to enhance agricultural productivity and for mitigation of climate change. To quantify SOC effects on productivity, process models serve as a robust tool to keep track of multiple plant and soil factors and their interactions affecting SOC dynamics. We used soil-plant-atmospheric model viz. DAISY, to assess effects of SOC on nitrogen (N) supply and plant available water (PAW) under varying N fertilizer rates in winter wheat (*Triticum aestivum*) in Denmark. The study objective was assessment of SOC effects on winter wheat grain and aboveground biomass accumulation at three SOC levels (low: 0.7% SOC; reference: 1.3% SOC; and high: 2% SOC) with five nitrogen rates (0–200 kg N ha^-1^) and PAW at low, reference, and high SOC levels. The three SOC levels had significant effects on grain yields and aboveground biomass accumulation at only 0–100 kg N ha^-1^ and the SOC effects decreased with increasing N rates until no effects at 150–200 kg N ha^-1^. PAW had significant positive correlation with SOC content, with high SOC retaining higher PAW compared to low and reference SOC. The mean PAW and SOC correlation was given by PAW% = 1.0073 × SOC% + 15.641. For the 0.7–2% SOC range, the PAW increase was small with no significant effects on grain yields and aboveground biomass accumulation. The higher winter wheat grain and aboveground biomass was attributed to higher N supply in N deficient wheat production system. Our study suggested that building SOC enhances agronomic productivity at only 0–100 kg N ha^-1^. Maintenance of SOC stock will require regular replenishment of SOC, to compensate for the mineralization process degrading SOC over time. Hence, management can maximize realization of SOC benefits by building up SOC and maintaining N rates in the range 0–100 kg N ha^-1^, to reduce the off-farm N losses depending on the environmental zones, land use and the production system.

## Introduction

Soil organic carbon (SOC) supports multiple soil functions determining soil physical, chemical and biological quality parameters (Reeves, 1997; Pan et al., 2009) contributing to the productive capacity of soils for food, fodder, and energy production (Lal, 2004). A number of factors influence SOC stocks and flows, spatially and temporally, in an ecosystem due to climate, land use, soil management, and cropping systems (Canadell et al., 2007). Building up SOC stock through agricultural measures (e.g., cover cropping, residue incorporation, reduced tillage) can affect soil properties, soil water retention and nutrient storage, affecting the productive capacity of soils (Ingram et al., 2016; Paustian et al., 2016). Decomposition of SOC releases mainly N, which can increase crop yields where crop N supply is limited (Palmer et al., 2017). Maintenance or build-up of SOC will require regular inputs of organic matter (OM) into the soil as the mineralization process will continually deplete the SOC over time, especially in environmental zones, where soil moisture and temperature are conducive for the mineralization process. The other effects of increased SOC content are decrease in the bulk density (Chen et al., 2017; Palmer et al., 2017; Minasny and McBratney, 2018) and small increase in volumetric water holding capacity (Rawls et al., 2003). Due to these multiple effects, there is a great interest to quantify SOC effects in agro-ecosystems. SOC increase can have positive and negative effects (Palmer et al., 2017; Minasny and McBratney, 2018). Among the multiple SOC effects, crop productivity and soil water retention are the priorities of the farmers to maintain sustainable agro-ecosystems. As European arable cropping systems are estimated to lose 300 Tg C (10^12^) year^-1^ (Janssens et al., 2003), it is necessary to segregate the SOC effects on crop yields and soil water retention and their combined synergistic benefits on crop productivity. Hence, quantification of SOC effects on N supply, soil water retention and crop productivity under varying fertility production system provides a science-based evidence of SOC benefits for making management decisions by farmers.

Winter wheat is one of the most widely cultivated arable crops, and the assessment of SOC-productivity relationship can generate insights into wheat crop management at field scale (Hansen et al., 2000; Christensen et al., 2009). An earlier study assessed SOC effects in winter wheat agro-ecosystem in seven sites representing diverse soil types, SOC content, management and climate including Netherlands (Palmer et al., 2017) found that SOC benefits are tangible in N deficient wheat production systems, whereas the benefits disappear in wheat agro-ecosystems, with surplus N. To add to this body of knowledge, this study provided insights into SOC effects under a context-specific set of soil type, SOC content, management and climate regimes in Denmark. Further, this study provided additional value to the findings of Palmer et al. (2017) because the range of SOC used in our study (0.7–2% SOC) is different than the SOC considered in Netherlands (2.8% and 4.3% SOC). SMARTSOIL^1^ consortium had access to the SOC and agronomic data on winter wheat from a long-term field trial in Askov from 1929 to 2008 and the field data provided us a unique opportunity to carry out the calibration and validation of DAISY model, to assess the productivity and SOC dynamics under winter wheat cultivation over 80 years. Hence, the study objective was to determine winter wheat productivity at three SOC ranges viz. low: 0.7% SOC; reference: 1.3% SOC; and high: 2% SOC with five nitrogen rates (0–200 kg N ha^-1^) and plant available water (PAW) at the low, reference, and high SOC levels.

## Materials and Methods

### Long-Term Field Trial in Askov

The long-term trial site in Askov (LTE-Askov; 55°28′N, 09°06′E) was established in 1923 and cropping system was 4-year crop rotation cycle of winter wheat, root crop, spring cereal and grass-clover from 1929 to 2008. In the 0–0.20 m plow layer, SOC was 1.3% and sand, silt and clay contents were 76%, 13%, and 11%, respectively, and the bulk density at plow layer was 1.5 g cm^-3^ (Christensen et al., 2006). LTE-Askov treatments consisted of two treatments, viz: Askov_0N and Askov_1.5NPK, implemented in a 4-year crop rotation cycle. Askov_0N treatment received no input of farmyard manure, nitrogen (N), phosphorus (P), and potassium (K) and crop residues were removed and Askov_1.5NPK treatment received 150 kg N, 28.5 kg P and 131.4 kg K ha^-1^. The measured field data from Askov_0N and Askov_1.5NPK winter wheat plots for the period 1929–2008, were split into calibration dataset (1929–1969) and validation dataset (1970–2008).

### DAISY Model Initialization and SOC Simulations

To assess long-term SOC dynamics in arable production systems, process models serve as a robust tool to keep track of multiple plant and soil factors and their interactions affecting SOC dynamics. The soil-plant-atmospheric model, DAISY, was implemented, due to its robustness for simulation of SOC dynamics and crop productivity in diverse climatic and cropping systems (Abrahamsen and Hansen, 2000). DAISY is a dynamic and deterministic soil-plant-atmosphere system model with separate sub-models for crop growth, C and N dynamics, heat, soil water and fate of pesticide use (Abrahamsen and Hansen, 2000). In the model, OM is constituted by added organic matter (AOM), soil microbial biomass (SMB), and soil organic matter (SOM) pool. AOM and SMB constitute relatively fast and slow turnover pools, whereas SOM is split into three pools; inert (SOM3), fast (SOM2), and slow turnover pools (SOM1), characterized by fixed C:N ratios and first-order decomposition rate coefficients (Hansen et al., 1991). AOM constitutes plant residues, added organic fertilizer or compost, etc.; the SMB pool is driving the biodegradation process and SOM is the recalcitrant humus fraction. Soil C and N dynamics were modeled by assuming constant C:N ratios in each pool (Bruun et al., 2003). The SOM pool, at the start of the simulation period was initialized to a steady state by simulating the pre-experimental period for 10 years before the onset of the experiment (Bruun and Jensen, 2002).

Daisy model was implemented in two steps viz. calibration and validation steps. For calibration step, DAISY model inputs were soil, weather, and winter wheat management data from LTE-Askov. The soil data on sand%, silt%, and clay%, bulk density and 1.3% SOC (hereafter called the “reference”) was provided to the model. The weather data was retrieved from the weather database, a common database created by the SMARTSOIL project (see footnote 1) to share and store information on long-term trial sites in SMARTSOIL consortium. Where the weather data was missing, the missing data was generated by the LARS-WG 5 weather generator (Semenov and Barrow, 2002; Semenov and Stratonovitch, 2010) based on statistical characteristics of actual sample of available measured weather data from LTE-Askov. The winter wheat management data included land preparation, sowing, fertilization and harvesting dates and application timing of 0, 50, 100, 150, and 200 kg N ha^-1^. Every year, the winter wheat was sown on 20th September and harvested on 20th August in Askov_0N and Askov_1.5NPK plots. The same planting and harvesting schedule was followed during the simulation period to reduce the yield variability due to these two factors. The N rates of 0, 50, and 100 kg N ha^-1^ was applied on 15th March and the N rates of 150 and 200 kg N ha^-1^ was split into two equal doses. The two equal dose consisted of basal dose on 15th March (50% of application rate) and second dose on 25th April (50% of application rate) to coincide with the critical growth stages of winter wheat for maximum uptake of N. In order to accommodate the residual nitrogen effect after the preceding glass-clover in the 4-year crop rotation, the model was provided with nitrogen dose of 40 kg N ha^-1^ (Høgh-Jensen and Schjoerring, 1996) in Askov_0N and Askov_1.5NPK plots.

In order to assess SOC effects on winter wheat productivity, the validated DAISY model was run with 0.7% SOC (low) and 2% SOC (high) in addition to model run with 1.3% SOC during the calibration and validation steps. The low, reference, and high SOC levels reflected the spectrum of SOC levels in Danish soils from sandy to loamy soils and the N rate reflected the standard N application rate in winter wheat production in Denmark. Each SOC level (low, reference, and high) was simulated under five different N rates. Each simulation run sequence consisted of an initial 10 years of the pre-experimental period followed by simulation of low, reference, and high SOC content under five N rates (0–200 kg N ha^-1^) for the period 1929–2008. The initial 10-year run was included in every simulation run to stabilize the treatment effect to a steady state. Each simulation was carried out with low, medium, and high SOC and the same SOC was used for the entire simulation period (1970–2008), which provided the SOC trends over the years during the simulation period. However, after each year of simulation, the same management practice was reset into the model with same dates for land preparation, sowing, fertilization, and harvest dates in each year, during the entire simulation period. Each cycle of wheat production starts with land preparation on 01 September, consisting of plowing the field, followed by seedbed preparation, sowing, fertilizer application, and harvesting.

### Pedotransfer Functions (PTF) for Determination of Plant Available Water (PAW)

To assess the SOC effects on PAW (m^3^ m^-3^), the correlation between the PAW and SOC was derived by regressing PAW contents at the low, reference, and high SOC contents. LTE-Askov soil data on clay%, silt%, OM%, and bulk density were used in PTF functions to derive saturated moisture content (𝜃_s_), residual moisture content (𝜃_r_), van Genuchten curve-fitting parameter α (1/cm = α) and van Genuchten curve-fitting parameter *n* and *m* = 1 – 1/*n* (Wösten et al., 2001). Four different PTF functions calculated the PAW to compare the differences and improve the reliability in estimation of hydraulic properties. The PTF functions were (a) HYPRES (Wösten et al., 1999), (b) hydraulic properties calculator (HPC) (Saxton and Rawls, 2006), (c) Rosetta model (Schaap et al., 1998), and (d) Danish PTF (Borgesen and Schaap, 2005). HYPRES PTF functions were developed based on 5,521 soil horizon profiles from different countries in Europe (Wösten et al., 1998) whereas HPC was developed with data from 1,722 United States soil samples (Saxton et al., 1986). The Rosetta model was built on the United States soil database whereas the Danish PTF is based on 3,226 soil samples from Denmark (Borgesen and Schaap, 2005). Subsequently, soil water content was calculated at different soil water potentials (kPa) by van Genuchten–Mualem model (VGM) (Vereecken et al., 2010), and PAW was considered as the difference in soil water content between the wilting point and the field capacity. We defined field capacity at 10 kPa and wilting point at 1,500 kPa and the difference of soil water content between the field capacity and the wilting point was taken as PAW.

### Model Calibration, Validation, and Statistics

**FIGURE 1:**
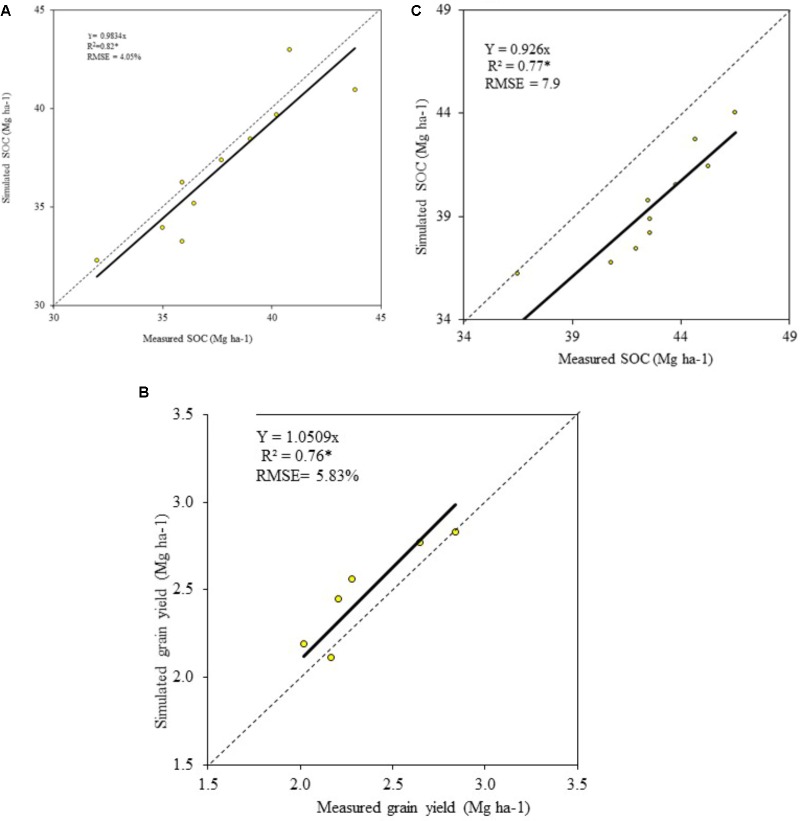
Validation of **(A)** SOC in Askov_0N, **(B)** winter wheat grain yield in Askov_0N, and **(C)** SOC in Askov_1.5NPK plots.

The model validation was carried out with MODEVAL 2.0 (Smith et al., 1997) by comparative plotting of measured and simulated SOC content in 0–0.20 m soil profile over 1970–2008 in winter wheat plots in Askov_0N (**Figure 1A**, RMSE = 4.05%) and Askov_1.5NPK treatments (**Figure 1C**, RMSE = 7.9%) and grain yields in Askov_0N (**Figure 1B**, RMSE = 5.83%). Measured SOC and grain yields were available every 4 years (4-year crop rotation) and so, 10 measured values were available for 1970–2008 period and the corresponding simulated values from the same 1970–2008 period were used for validation (**Figures 1A–C**). ANOVA tests were run on to assess effect of SOC, N and SOC × N on winter wheat grain and aboveground biomass yields at low, reference, and high SOC under 0–200 kg N ha^-1^. The standard error and LSD_0.05_ of the simulated values were calculated in MS excel using the data analysis tool pack and significant effects are denoted as ^∗∗∗^*P* < 0.001, ^∗∗^*P* < 0.01, ^∗^*P* < 0.05, ns, non-significant.

## Results

### Validation of SOC Dynamics, Winter Wheat Productivity, and PAW

The modeled and the measured values of SOC in 0–0.20 m soil profile in Askov_0N (**Figure 1A**, *R*^2^ = 0.82^∗^ and Askov_1.5NPK treatments (**Figure 1C**, *R*^2^ = 0.77^∗^), had significant positive correlation coefficient. Similarly, significant positive correlation coefficient was obtained for winter wheat grain yields in Askov_0N (**Figure 1B**, *R*^2^= 0.76^∗^). The validation on SOC dynamics to 0.20 m soil depth under fertilized (Askov_1.5NPK, RMSE = 7.9%) and non-fertilized treatments (Askov_0N, RMSE = 4.05%) demonstrated that DAISY was robust in simulation of winter wheat productivity at the tested SOC range under 0–200 kg N ha^-1^ treatments. DAISY model has been used in Denmark to quantify soil water balance (Salazar et al., 2013) and SOC (Bruun et al., 2003). This provided the scientific rationale for using DAISY for simulation of grain yields and aboveground biomass (grain + straw) accumulation at low, reference, and high SOC content under 0–200 kg N ha^-1^ treatments in this study.

The long-term change dynamics of SOC, presents challenges to simulate the SOC dynamics over time due to unavailability of data for calibration and validation of models. In this regard, we had unique access to LTE-Askov data and simulation window of 80 years (1929–2008) to assess the long-term change dynamics of SOC and triangulate the field data with simulated data and its effects on agronomic productivity. We chose DAISY, due to its robustness to keep track of the SOC flows and stocks in the soil, taking account of the plant and the management factors. We validated the DAISY model SOC and grain yield outputs with measured data from Askov_0N and Askov_1.5NPK. The model considers only N as the limiting factor and the grain yields and aboveground biomass are not affected by P and K inputs. In similarity to our study, DAISY model had been used for simulation of crop grain yield and aboveground biomass accumulation in several model comparison exercises (Dewilligen, 1991; Vereecken et al., 1991; Diekkrüger et al., 1995) and validation of crop yield in winter wheat in three sites in the Netherlands (Hansen et al., 1991). In a comparison of nine SOM models to assess management effects (land use, fertilizer, manure, and rotation treatments) on SOC dynamics in seven LTEs in diverse climatic gradients, DAISY outputs were comparable, with a similar margin of error among other models (DNDC, RothC, CENTURY, CANDY, NCSOIL) and even better than the SOMM, ITE, and Verberne models (Smith et al., 1997). This provides a scientific rationale for use of DAISY model to assess SOC dynamics.

### Trend Comparisons of Measured and Modeled SOC Data

**FIGURE 2:**
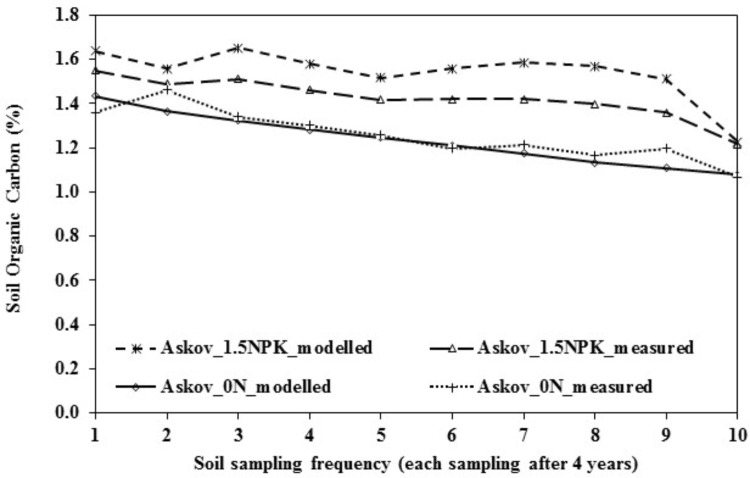
Comparison of modeled and measured SOC values in Askov_0N and Askov_1.5NPK plots. Sampling frequency is 4 years and the timeline of sampling period is 40 years.

The measurement of the SOC at the experimental site started in 1923 in Askov_0N and in 1929 in Askov_NPK plot. In 1923, Askov_0N plot had 1.6% SOC content, which decreased to 1.4% by 1969 and to 1.1% by 2008. Similarly, the Askov_1.5NPK had 1.8% SOC in 1929 and it reduced to 1.5% by 1969 and to 1.2% by 2008. Bulk density measurements remained the same throughout the measurement period and so the changes in SOC was due to continuous removal of the crop residues and decomposition of the available SOC in the soil. The measured and the modeled SOC values during the calibration period (1969–2008) showed a similar trend for Askov_0N and Askov_1.5NPK (**Figure 2**) and the correlation between the measured and modeled SOC and grain yield values are provided in **Figure 1A** (SOC), **Figure 1B** (grain yield), and **Figure 1C** (SOC). The measured SOC value provided from the experimental site showed that the SOC range used for simulation is achievable in the soil and climate conditions at the experimental site.

### SOC and N Effects on Winter Wheat Grain Yield and Aboveground Biomass (Grain + Straw) Accumulation

Soil organic carbon levels and N rates had significant effects (*P* < 0.001) on winter wheat grain yields and aboveground biomass accumulation. In similarity, SOC × N effects were significant (*P* < 0.01) for grain yields and aboveground biomass accumulation. The SOC × N interactions implied that the SOC level effects differed at varying N application rates from 0 to 200 kg N ha^-1^.

With 0 kg N ha^-1^, the winter wheat grain yield increased significantly by 0.28 Mg ha^-1^ from low to reference SOC content and by 0.30 Mg ha^-1^ from reference to high SOC content (**Table 1**). Hence, the increase in grain yields from low to high SOC content was 0.58 Mg ha^-1^, 31% increase in grain yield, which was a significant improvement in grain yields over the low SOC content. Similarly, at 0 kg N ha^-1^, the aboveground biomass (straw + grain) increased significantly by 0.59 and 0.63 Mg ha^-1^ from low to reference and reference to high SOC levels, respectively.

**Table 1:** Winter wheat grain and aboveground biomass (grain + straw) yields (mean ± standard error) at low, reference, and high SOC at 0–200 kg N ha^-1^.

N rate/SOC	Low SOC (0.7%)		Reference SOC (1.3%)		High SOC (2%)		*LSD_0.05_*	
Kg N ha^-1^	Grain Mg ha^-1^	Grain + straw Mg ha^-1^	Grain Mg ha^-1^	Grain + straw Mg ha^-1^	Grain Mg ha^-1^	Grain + straw Mg ha^-1^	Grain	Grain + straw
0	1.88 ± 0.06	4.18 ± 0.11	2.16 ± 0.06	4.77 ± 0.12	2.46 ± 0.07	5.40 ± 0.14	0.18	0.36
50	2.27 ± 0.28	6.55 ± 0.62	3.13 ± 0.26	8.26 ± 0.35	3.98 ± 0.25	9.64 ± 0.17	0.82	1.47
100	4.13 ± 0.45	10.03 ± 0.49	5.30 ± 0.32	11.35 ± 0.21	6.10 ± 0.15	12.20 ± 0.14	1.14	1.11
150	6.06 ± 0.14	12.16 ± 0.36	6.67 ± 0.25	12.80 ± 0.42	6.67 ± 0.25	12.80 ± 0.42	0.78	1.39
200	6.67 ± 0.25	12.80 ± 0.42	6.67 ± 0.25	12.80 ± 0.42	6.67 ± 0.25	12.80 ± 0.42	0.89	1.46

At 50 kg N ha^-1^, the winter wheat grain yield increase was significant, with increase of 0.86 and 0.85 Mg ha^-1^ from low to reference and reference to high SOC, respectively (**Table 1**), whereas aboveground biomass increase was significant only from low to reference SOC. Similarly, at 100 kg N ha^-1^, the grain yield and aboveground biomass increase was significant by 1.17 Mg ha^-1^ and 1.32 Mg ha^-1^, respectively, from low to reference SOC. At 150 kg N ha^-1^ and 200 kg N ha^-1^, there was no significant increase in grain yield and aboveground biomass between low, reference, and high SOC contents.

In summary, there was relatively higher SOC effects on both grain yields and aboveground biomass at 0–100 kg N ha^-1^ and the effects decreased with increasing N rates until there was no SOC effects at 150–200 kg N ha^-1^. With higher SOC content, lower N rate is required to attain a locally relevant yield ‘plateau’ compared to the soils with lower SOC content and in contrast, higher N rate will be required to attain the same yield ‘plateau’ with lower SOC content.

### SOC Effects on Plant Available Water (PAW)

**FIGURE 3:**
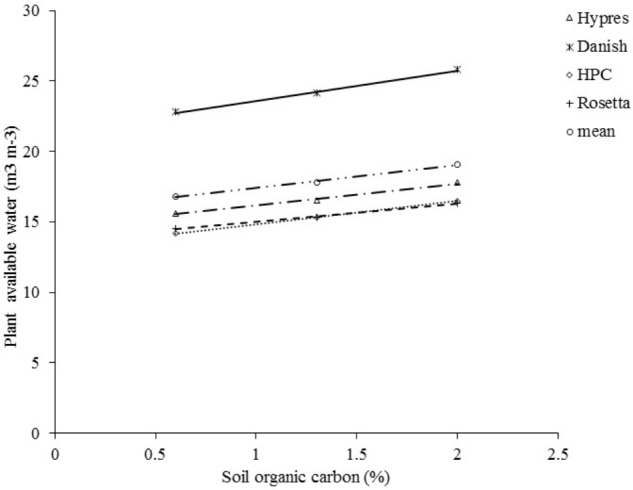
Relationship between plant available water content and SOC at 0.7%, 1.3%, and 2% SOC at LTE-Askov in Denmark. All four PTF functions exhibited linear relationship given by *Y* = 0.945*x* + 14.52, *R*^2^ = 0.99^∗∗∗^ (Hypres), *Y* = 1.309*x* + 21.319, *R*^2^ = 0.99^∗∗∗^ (Danish), *Y* = 0.991*x* + 13.109, *R*^2^ = 1^∗∗∗^ (HPC), and *Y* = 0.783*x* + 13.617, *R*^2^ = 0.99^∗∗∗^ (Rosetta).

A highly significant positive correlation between PAW and SOC was obtained with Danish PTF, given by *Y* = 1.3094*x* + 21.319 (*R*^2^ = 0.99^∗∗∗^) (*Y* = PAW and *X* = SOC) (**Figure 3**). The measured PAW at LTE-Askov was 21%, in close proximity to calculated value of 23%, demonstrating the robustness and reliability of the Danish PTF to predict PAW, validating the highly positive correlation between SOC and PAW.

Soil organic carbon and calculated PAW content had significant positive correlation (*R*^2^ = 0.99–1^∗∗∗^), and higher SOC content retained correspondingly higher PAW in LTE-Askov soils (**Figure 3**). The PAW calculated by VGM, based on generated hydraulic parameters by the four PTFs, demonstrated a similar trend of significant positive correlation between SOC and PAW. PAW based on Danish PTF (Borgesen and Schaap, 2005) resulted in highest calculated PAW content compared to the three other PTFs (HPC, Rosetta, and HYPRES). HYPRES PTF calculated the second highest PAW content followed by HPC and Rosetta PTFs. When the mean of PAW was averaged across the four PTFs at different SOC contents, there was positive correlation between PAW and SOC given by the linear relationship PAW (%) = 1.007 × SOC (%) + 15.641.

The regression relationship showed that the increase in PAW within the tested SOC range (0.7–2% SOC) was only 1.4%, which is not large enough change to affect yields. The effect of such a small change in PAW is difficult to verify in the field and is unlikely to have any significant change in yields and biomass accumulation. Hence, PAW did not have any significant role in yield and aboveground biomass accumulation.

## Discussion

### SOC and Provision of Ecosystem Services

Soil organic carbon affects multiple ecosystem services, and SOC build-up and management can pose different challenges depending on the environmental zones and the context-specific production systems (Palm et al., 2014). SOC maintenance is a challenge, as the mineralization processes continuously degrade SOC over time. Where the arable farming systems are integrated with livestock, the manure from livestock are good sources of OM to build up SOC whereas it can be a challenge in other arable production systems unless dedicated practices like cover crops, no-till or mulch farming are practiced to replenish the SOC (Lehtinen et al., 2014). SOC can have both positive and negative effects, and the management have huge influences on the benefits from SOC. Under N non-limiting wheat production systems, nutrients released through the decomposition of the SOC especially mobile N can leach beyond the root zone and pollute the groundwater, contaminating the water supply for human consumption (Palmer et al., 2017). The losses of N downstream can induce algal bloom and eutrophication, which can have devastating impacts on the aquatic and other fish species. In addition, some of the nitrogen forms can be lost as nitrous oxides, which have global warming potential of 300 times more than the carbon dioxide (Burgin et al., 2013). In contrast, under N deficient wheat production systems, building and maintaining SOC can provide wider benefits with provision of multiple ecosystem services like supply of macro and micronutrients, carbon sequestration, food and fodder production, mitigation of soil erosion and support habitat for biodiversity (Ghaley et al., 2014). Hence, the benefits accrued from SOC increase is evident only in N deficient wheat production system, an important management decision for the wheat producing farmers.

### SOC Effects on Grain Yield and Aboveground Biomass

The range of SOC values used for the simulation at the LTE-Askov is within the ranges reported for the trial site, as evident from the measured SOC values in Askov_0N and Askov_1.5NPK plots (LTE-Askov, **Figure 2**). Similar SOC range of 1.2–1.7% was reported from another study at the same trial site (Thomsen and Christensen, 2004). The reduction of SOC content during the experimental period was attributed to decomposition of the SOC releasing N and other macro and micronutrients, and the SOC effects are only transient if efforts are not put into replenishment of OM to maintain the SOC stock.

Our study demonstrated that, at N application rates of 0–100 kg N ha^-1^, SOC had benefits in terms of enhancing winter wheat productivity (**Table 1**). Similar positive correlations in grain yield-SOC relationships were reported in several field studies (Thomsen and Christensen, 2004; Persson et al., 2008; Seremesic et al., 2011; Yang et al., 2011; Mikanova et al., 2012) and one simulation study across seven sites and pedo-climatic zones (Palmer et al., 2017). In our simulations, winter wheat grain and straw yields increased with increases in SOC (low, reference, and high), which is supported by findings from another field experiment at LTE-Askov, where increased SOC increased spring barley yields (Christensen et al., 2009). Spring barley grain and straw yields increased with increase in SOC indicating a positive relationship between SOC and yield (Christensen et al., 2009) in conformity to our study. However, application of more than 90 kg N ha^-1^ nullified the SOC effects on grain and straw yield (Christensen et al., 2009) which conforms to our decreasing SOC effects on winter wheat with increasing N fertilizer with significant effect only up to 100 kg N ha^-1^ (**Table 1**). The benefits of SOC on spring wheat grain yields was reported from a 24 years trial at Jyndevad in Denmark, where an N substitution rate of 15–27 kg N ha^-1^ was attained with long term catch crops building up higher SOC in the soil (Hansen et al., 2000). This increase in yield is similar to the winter wheat yields in our study, where significant increase in grain and biomass yields were obtained with increasing SOC content at 0–100 kg N ha^-1^ (**Table 1**), providing evidence of SOC × N effects on winter wheat productivity. Higher SOC content had a significant influence on grain yield and aboveground biomass increase only at 0–100 kg N ha^-1^, which demonstrated the benefits of building up SOC to compensate for the fertilizer N inputs. This indicated that the maximum SOC benefits can be realized only at 0–100 kg N ha^-1^ input under the Danish wheat production agro-ecosystems and the benefits were non-existent as the N rates are increased to more than 100 kg N ha^-1^ due to N losses into groundwater, eutrophication and algal blooms downstream and nitrous oxide losses as greenhouse gas. Hence, SOC benefits are contextual and multiple benefits are only realized in N deficient wheat production systems.

### SOC Effects on PAW (m^3^ m^-3^)

Our study demonstrated that the four PTFs are robust enough to predict PAW based on the minimum soil parameters collected in the field trials, which can provide insights into water availability (m^3^ m^-3^) in the soil. In line with our study, significant positive correlations (**Figure 3**) between SOC and PAW, have been reported in other studies in volumetric (Rawls et al., 2003) and gravimetric basis (Emerson, 1995) under diverse environments (Bationo et al., 2013) including a study (gravimetric) on 41 Danish soils (Resurreccion et al., 2011). A study in North Dakota in sandy, medium and fine textured soils demonstrated that soils with higher SOC retained more soil water (gravimetric) irrespective of the soil types (Bauer and Black, 1992) supporting the outcome of this study that SOC has positive effects on soil water retention. An exhaustive investigation of soil type-PAW correlation, based on the soil samples collected from across United States (Hudson, 1994), demonstrated a significant positive SOM-PAW correlation (volumetric) across three soil types (sandy, silty clay, and silty loamy clay), in line with our findings. Some recent studies (Palmer et al., 2017; Minasny and McBratney, 2018) also reported SOC positive effects on PAW in line with our study. Hence, our study supports the positive relationship between SOC and PAW. However, the PAW increase was too small to affect the crop yields and aboveground biomass accumulation. Moreover, the underlying mechanisms of SOC-PAW relationship need to be further explored.

## Conclusion

The benefits of SOC can be positive and negative and maintenance of SOC will require regular inputs of OM into the soil. The efforts to maintain the SOC and reap the benefits, are contextual depending on the land use, environmental zones, and management practices. In our study, increasing SOC content had significant positive effects on winter wheat grain yield and aboveground biomass at only 0–100 kg N ha^-1^ and the SOC effects were non-significant with increasing N inputs at 150–200 kg N ha^-1^. SOC and PAW were positively correlated but the increase in PAW was minimal with no significant effects on grain yields and aboveground biomass accumulation. Our study findings were similar to other studies (Minasny and McBratney, 2018) carried out in diverse environments (Palmer et al., 2017), which lends credence to this study in confirming that the earlier results from Netherlands and other six sites were equally applicable in Denmark and other relevant environments. In order to improve our analysis, future investigations should include quantification of dis-benefits viz. N leaching, N loss downstream and nitrous oxide loss, to provide additional insights into the extent of dis-benefits with increasing N input. Hence, benefits and dis-benefits parameters need to be measured in future studies in order to generate a complete analysis of SOC effects for improved management decision by farmers, agricultural advisors and policy makers.

## Author Contributions

BG wrote the first draft and made the subsequent revisions of the paper with data inputs from HW, JO, JY, and PS. KS, SB, PK, J-PL, and JP reviewed the document and provided inputs to improve the scientific content of the manuscript. RF and MB helped with cleaning the weather data and CB and YK helped with setting up the DAISY model for carrying out simulations.

## Conflict of Interest Statement

The authors declare that the research was conducted in the absence of any commercial or financial relationships that could be construed as a potential conflict of interest.

## Footnotes

1 http://smartsoil.eu/
